# Translation of a physiologically‐based pharmacokinetic model for dabigatran etexilate to the design of a safety and efficacy study in post‐partum women

**DOI:** 10.1002/bcp.70344

**Published:** 2025-11-21

**Authors:** Kayode Ogungbenro, Lorna Aucott, Farhad Kamali, Paul Ayuk

**Affiliations:** ^1^ Centre for Applied Pharmacokinetic Research, Division of Pharmacy and Optometry, School of Health Sciences, Faculty of Biology, Medicine and Health University of Manchester Manchester UK; ^2^ Insititute of Medical Sciences University of Aberdeen Aberdeen UK; ^3^ Translational and Clinical Research Institute Newcastle University Newcastle upon Tyne UK; ^4^ Newcastle upon Tyne Hospitals NHS Foundation Trust UK

**Keywords:** dabigatran, modelling, pharmacokinetics, post‐partum women

## Abstract

**Aims:**

To translate a PBPK model developed for the direct oral anticoagulant, dabigatran etexilate, the prodrug of dabigatran, based on data obtained from healthy men to healthy non‐pregnant, pregnant and post‐partum women. To evaluate safety and efficacy of dabigatran etexilate in post‐partum women using simulations and design a future clinical study to support model verification.

**Methods:**

An integrated PBPK/PD model in healthy non‐pregnant, pregnant and post‐partum women was translated from the PBPK model in healthy men by capturing physiological changes. The model was also linked to established exposure‐response relationships for coagulation indices and plasma‐breast milk transfer using data from a pilot study. Finally, the model was used for simulation and the design of a prospective clinical study.

**Results:**

The model predicted comparable plasma concentration profiles between healthy men and healthy, pregnant and post‐partum women, by capturing their physiological differences. The model also captured plasma concentration and activated partial thromboplastin time (a coagulation index) data from a pilot study in post‐partum women available in the literature. A sample size of 20 and 50–60 were determined to be appropriate for safety and efficacy arms respectively, of a clinical trial for model verification.

**Conclusion:**

An integrated PBPK/PD model for dabigatran etexilate in post‐partum women has been developed successfully. The model was used to design a future clinical trial examining safety and efficacy of dabigatran in postpartum women at risk of thromboembolism.

## Graphical abstract

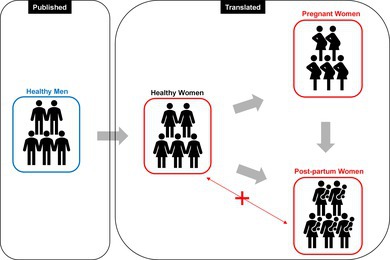

**What is already known about this subject**

- Direct oral anticoagulants, including dabigatran etexilate, a prodrug for dabigatran, have revolutionised thromboprophylaxis treatment.
- PBPK modelling can be used to evaluate the risks and benefits associated with drugs in pregnant and post‐partum women, and to support clinical study design.
- A PBPK model has been developed for dabigatran etexilate/dabigatran in healthy men.

**What this study adds**

- PBPK model in healthy men was translated to healthy non‐pregnant, pregnant and post‐partum women by accounting for physiological changes.
- An integrated PBPK/PD model that accounts for plasma concentration, coagulation indices and plasma‐breast milk transfer in post‐partum women was developed.
- The model predicted very low dabigatran concentrations in breast milk in post‐partum women which in turn suggests a very low risk to the suckling child.

## 1 INTRODUCTION

Post‐partum women are at high risk of venous thrombo‐embolism (VTE) compared to nonpregnant women, with pregnancy increasing the risk of VTE by up 5‐fold.^1^ In the United States, VTE was reported to be responsible for approximately 9% of deaths in post‐partum women.^2^ While direct oral anticoagulants (DOACs) have revolutionised thromboprophylaxis treatments in different populations, daily heparin injection remains the only option available in postpartum women due to safety concerns associated with secretion of DOACs into breast milk which could harm the suckling child. In the last ten years, there has been increased awareness to address the unmet need of using drugs in pregnant and postpartum women safely. Regulatory authorities and interest groups have released guidance documents on best practices for conducting studies safely and efficiently in this population.^3^, ^4^, ^5^

Dabigatran etexilate (DABE) was first licensed nearly 15 years ago as the first DOAC, 50 years after the advent of warfarin, and it is currently licensed for use in adults and children, but not in pregnant and post‐partum women.^6^, ^7^ DABE is a prodrug and is completely converted to the active agent dabigatran following oral administration with an oral bioavailability of around 7%, and only trace amounts of DABE are detected in plasma.^8^, ^9^, ^10^ Since dabigatran is highly polar, it is anticipated that the drug does not readily transfer into breast milk nor is absorbed from the gut of the suckling baby. This makes DABE an ideal candidate for thromboprophylaxis during breastfeeding. Recently, in a pilot study it was reported that dabigatran is secreted into breast milk in very low concentrations which are 100 000 times below levels needed to alter coagulation indices in term and pre‐term neonates.^11^

Dose optimisation in post‐partum women is challenging, because immediately after delivery various pregnancy associated physiological changes are still evident, before they gradually return to non‐pregnancy state over time. Physiologically based pharmacokinetic (PBPK) modelling is a tool that can be used to evaluate the risks and benefits associated with drug use in pregnant and post‐partum women.^12^ It can be used to support study design and to further explore pharmacokinetic and pharmacodynamic (PK/PD) relationships in these understudied populations, with the aim of dose optimisation.

The aim of this study was to translate the DABE‐dabigatran PBPK model recently developed and validated in healthy male volunteers to post‐partum women by accounting for physiological changes in this population.^13^ This model is expected to capture drug transfer between blood/plasma and breast milk, and exposure‐response relationships by incorporating coagulation indices: activated partial thromboplastin time (aPTT), diluted thrombin time (dTT), and ecarin clotting time (ECT). The final PBPK/PD analysis will use modelling to leverage available limited data, for simulation and design of a future safety and efficacy study.

## 2 METHODS

### 2.1 PBPK model in healthy men

Lang *et al* ^13^ developed and verified a reduced joint oral DABE‐dabigatran PBPK model using data from healthy male volunteers, which accounted for regional distribution of P‐glycoprotein (P‐gp) in the intestine, an important consideration in the absorption of DABE and dabigatran. The model development was based on clinical data from a wide range of doses; from micro to therapeutic doses and the model verification was based on clinical data from drug–drug interaction (DDI) studies that used a range of P‐gp inhibitors with DABE as a probe substrate. The physiological parameters of this model were based on typical adult values, which included body weight, cardiac output, organ blood flows derived from fractions of total cardiac output and organ volumes derived from fractions of organ weights and densities. Drug specific parameters were either from *in vitro* published studies or optimised using intravenous clinical data. Detailed descriptions of the equations for this model are in Lang *et al*.^13^

### 2.2 PBPK model in healthy non‐pregnant, pregnant and post‐partum women

In this study, the DABE‐dabigatran PBPK model previously developed in healthy men was translated to healthy non‐pregnant, full‐term pregnant and post‐partum women. The translation of the model from healthy men was based on adjustments for various physiological changes that occur in the different population groups, using data obtained from the literature. In each population of women, key significant differences included changes to total body weight and organ weights, total cardiac output and organ blood flows, plasma volume and albumin level (Table 1 and Supplementary Tables S1 and S2). In the case of pregnant women, these also included changes associated with presence of foetus and related organs/tissues at full term. However, in the case of post‐partum women, it was assumed that immediately after delivery, the physiological state of women is akin to full term pregnancy, therefore, relevant physiological parameters for post‐partum women were assumed to be similar to that of pregnant women. Drug specific parameters were kept mostly as values in healthy men (Supplementary Table S3).

**TABLE 1:** Summary of key physiological parameters used in the PBPK models for healthy men and healthy non‐pregnant, pregnant and post‐partum women (obtained from Valentin, 2002^14^).

Parameter	Healthy men	Healthy non‐pregnant women	Pregnant women	Post‐partum women
Total body weight (kg)	70	60	72.5	66
Total blood volume (L)	5.3	3.9	5.35	4.85
Cardiac output (L/min)	6.5	5.9	7.3	7.6
Albumin (g/L)	46	43	31.1	31.1
Glomerular filtration rate (ml/min)	125	110	150	150

### 2.3 PBPK/breast milk model

Using data from a pilot study of a single dose of 220 mg dabigatran in two post‐partum women,^11^ an empirical model from the literature was used to describe drug transfer between plasma and breast milk in these individuals.^15^ The study was conducted with the informed consent of participants; post‐partum women were approached two to seven days after delivery, only if they made an informed decision not to breastfeed their babies. The study protocol was reviewed and approved by the National Research Ethics Service‐Committee North East, Newcastle & North Tyneside, United Kingdom, Ref: 15/NE/0331). This empirical dabigatran plasma/breast milk model was based on a one compartment model for each of plasma and breast milk (Supplementary Figure S1). The transfer of unbound drug between plasma and breast milk was captured with a parameter, ${\mathrm{CL}}_{M}$, which was assumed to be the same in both directions, and constant during breast feeding. Currently, there is no information in the literature that suggests that dabigatran is substrate for a known transporter for drug secretion in the breast milk. Parameter fitting was carried out using a naïve pooling approach in NONMEM, 7.4.3.^16^ A description of this model and the estimated parameters are presented in the Supplementary Material (Table S4). The established drug transfer between plasma and breast milk with this model was subsequently connected to the translated PBPK model for port‐partum women for simulation of plasma and breast milk concentrations. This model can therefore be used to simulate drug concentration in breast milk and by assuming a consumption rate for an infant, the dose of the drug during feeding can be determined; this can be obtained as infant daily dose ( IDD).^3^ Two consumption rates of 150 and 200 mL/kg/day breast milk were explored for a new born infant with an average weight of 3.5 kg.^3^ Also, the amount of dabigatran consumed daily by the infant relative to the ingested equivalent dose by the mother, in percentage (percentage relative infant daily dose – %RIDD) was determined.^3^

### 2.4 PBPK/PD model simulations

To explore PK/PD relationships, the PBPK models in different populations were combined with the population PK/PD models for coagulation indices: aPTT, dTT and ECT. In various clinical studies, these indices have been validated as PD surrogate endpoints for measurement of the anticoagulant effect of dabigatran, and were employed in the clinical development of DABE.^7^ Using individual plasma and aPTT, dTT and ECT data in adults and children at different dose levels, population PK/PD models have been established.^7^ It was shown that there is direct correlation between dabigatran plasma concentrations and the anticoagulation effects as measured by aPTT, dTT and ECT. In the case of dTT and ECT, these were described by linear relationships, however the relationship was found to be nonlinear for aPTT. These models were used to support licensing of DABE in adults, and dose recommendations in special populations, e.g., children and renally impaired adults.^6^, ^7^ These established PD models were therefore connected to the PBPK models for DABE‐dabigatran, to obtain PBPK/PD models. Descriptions of the models and parameters estimates used are presented in the Supplementary Material (Tables S5–S7).

### 2.5 Integrated PBPK/PD model

Integrated models that linked the PBPK models for plasma concentration of DABE‐dabigatran to those for breast milk (post‐partum women only) and PD for aPTT, dTT and ECT were implemented in MATLAB, 2024a.^17^ These models were used to simulate plasma and breast milk concentrations, and to explore the link between dabigatran plasma concentrations and aPTT, dTT and ECT. 10 000 simulations were conducted in each scenario, the median and 95% prediction intervals were obtained and plotted for different populations, assuming a dosage regimen of 110 mg followed by 220 mg every 24 h for 10 days.

### 2.6 Proposed clinical study design and sample size calculations

The proposed prospective clinical study is based on two arms evaluating DABE safety and efficacy. The aim of the safety arm is to determine whether DABE dose taken by the mother is safe for the breastfed infant based on the concentration of the drug in breast milk and the amount ingested by the infant. This is expected to be a single dose study of 220 mg, which is the licensed dose for adults. The PBPK/PD model was therefore used to simulate drug concentrations in the breast milk to obtain expected %RIDD for sample size calculation. For this arm, the sample size is based on restricting the upper 95% confidence interval (CI) of %RIDD to be below a threshold, considered safe.

For the efficacy arm, the primary outcome is trough concentration ( $C_{\min}$) after two doses of DABE – 110 mg followed by 220 mg after 24 h. $C_{\min}$ represents an appropriate marker of efficacy because it has been shown that there is direct correlation between exposure and efficacy for anticoagulant effect of dabigatran. Given that $C_{\min}$ is often skewed, power calculations were based on log transformed estimates which were simulated by the PBPK/PD model. This is based on 95% CI of $C_{\min}$ greater than a threshold, to ensure adequate exposure over the dosing interval.

## 3 RESULTS

### 3.1 Integrated PBPK/PD model in healthy men and healthy non‐pregnant, pregnant and post‐partum women

**FIGURE 1:**
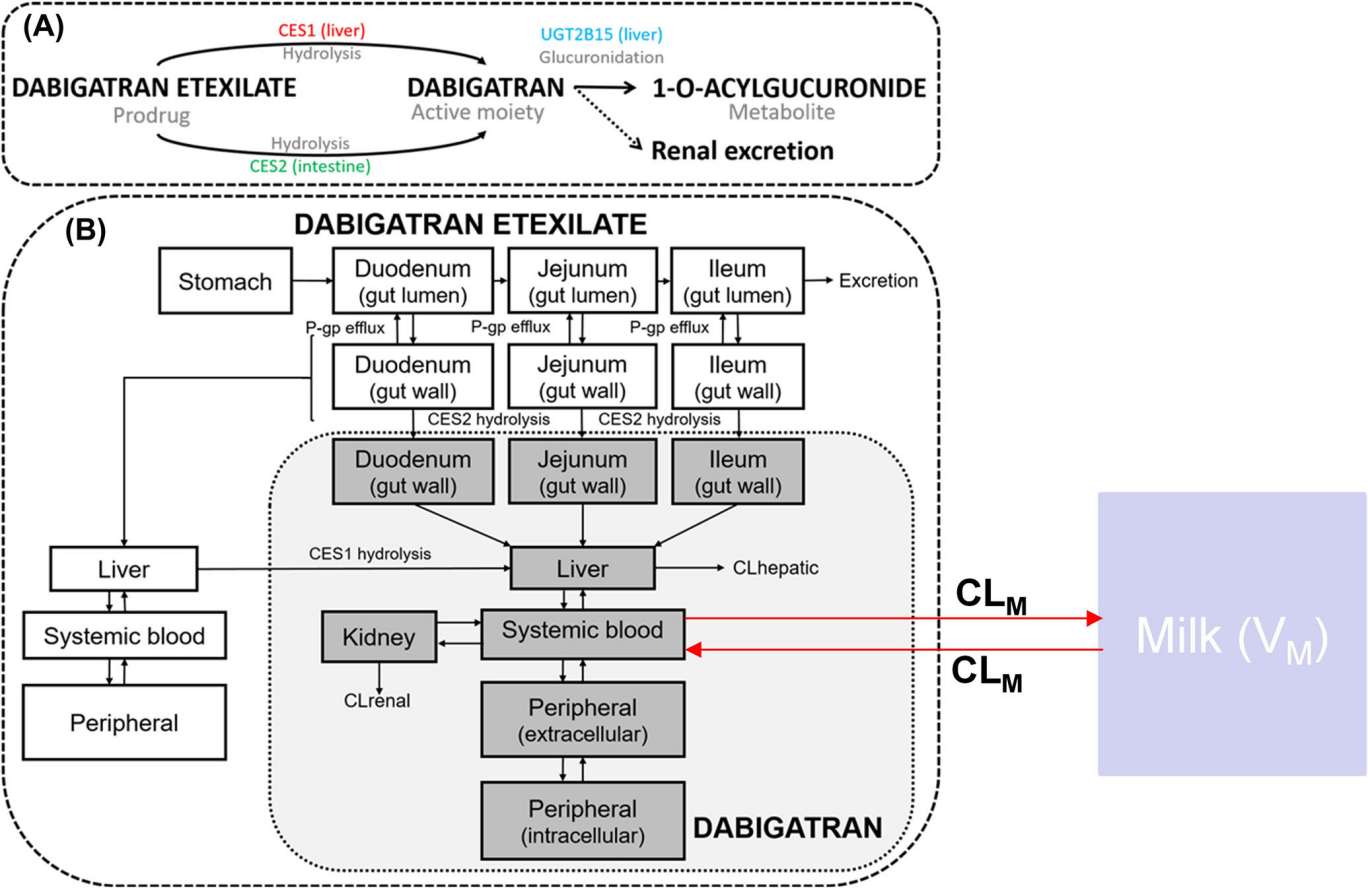
A schematic presentation of the PBPK model for plasma and breast milk concentrations in healthy men and healthy non‐pregnant, pregnant and post‐partum women (adapted from Lang et al, 2021,^13^ permission obtained).

The structure of the integrated model is shown in Figure 1; this describes the concentration in different tissues and breast milk in‐post‐partum women. The main parameter for drug transfer between plasma and breast milk was estimated using data from a pilot study.^11^ The parameter, ${\mathrm{CL}}_{M}$ was estimated to be equal to $0.0126\;L/h\;\left(\mathrm{RSE}=34\%\right)$, and it was assumed to be the same in both directions due to lack of data to support estimation of different parameters in both directions. This is similar to the mean of the estimates ( $0.0278\;L/h\;\left(\mathrm{RSE}=32\%\right)$ and $0.0032\;L/h\;\left(\mathrm{RSE}=39\%\right)$) of ${\mathrm{CL}}_{M}$ obtained for the two patients in the pilot study when the median predicted plasma concentration profiles from the PBPK model were used. Methods that predict milk‐to‐plasma (M/P) ratio using breast milk composition and physicochemical properties^18^, ^19^ were tried, but they did not capture the observed data adequately. The predicted plasma concentrations were also linked to coagulation indices to explore PBPK/PD effects.

The model was used to simulate 10 000 subjects in different populations (Supplementary Figure S2 shows the median profiles for plasma concentrations and coagulation indices). These profiles overlapped for PK and PD in different populations, which suggest that there are no significant differences between the profiles, mainly because the physiological changes implemented for different populations have not affected the PK of DABE‐dabigatran, and subsequently the PD. Also, in Supplementary Figure S3 are the plots of the medians and 95%PIs for healthy non‐pregnant and post‐partum women, which also overlapped for plasma concentrations and coagulation indices. Table 2 shows the summary statistic of $C_{\min}$ after the first, second and 10th (steady state) doses of DABE in different populations. These results showed that the $C_{\min}$ between different populations are comparable, although values for pregnant and post‐partum women are slightly lower compared to healthy men and non‐pregnant women. This is probably due to higher GFR; 20 and 36% higher GFR in post‐partum women relative to healthy men and non‐pregnant women respectively.

**TABLE 2:** A summary of trough concentration ( $C_{\min}$) after the first, second and 10th (steady state) doses of DABE.

Percentiles	Healthy men			Healthy non‐pregnant women			Pregnant women			Post‐partum women		
	1st dose	2nd dose	Steady state	1st dose	2nd dose	Steady state	1st dose	2nd dose	Steady state	1st dose	2nd dose	Steady state
2.5th	2.12	6.48	9.45	2.14	6.66	9.50	1.76	5.21	7.14	1.86	5.60	7.40
5th	2.55	7.73	11.14	2.57	7.99	11.36	2.03	6.13	8.34	2.20	6.61	8.68
50th	6.45	20.05	27.97	6.60	21.19	29.19	5.16	15.83	20.83	5.34	16.70	21.53
95th	17.93	54.56	74.11	18.78	60.28	81.78	15.32	45.78	58.54	14.59	46.10	58.20
97.5th	21.84	65.76	89.82	22.33	73.10	99.22	18.70	56.14	71.79	18.32	56.74	71.74

**FIGURE 2:**
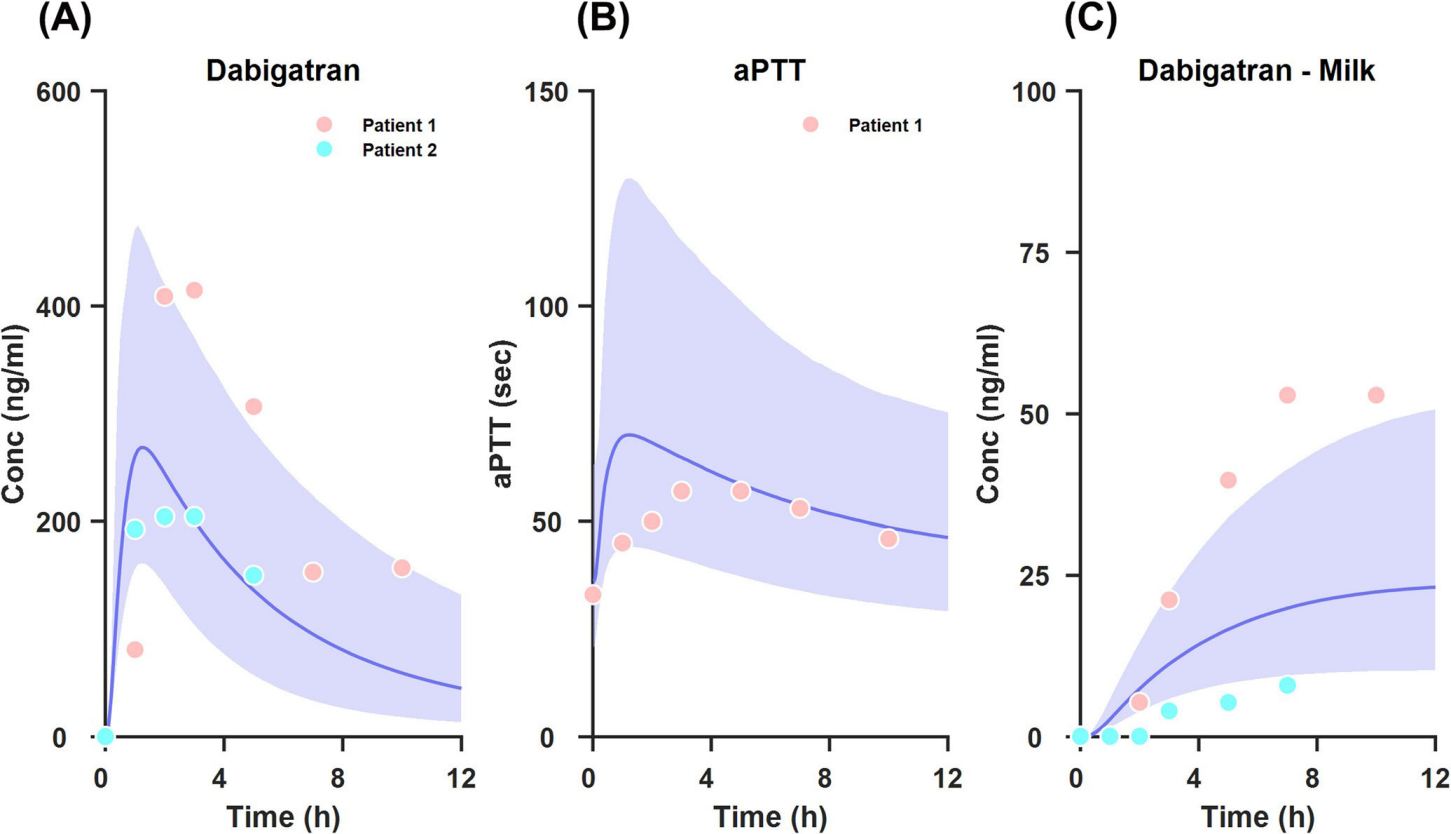
Observed data and simulated median lines, 95%PIs for dabigatran plasma concentration, aPTT and breast milk concentration in post‐partum women from pilot data and modelling following a single dose of DABE.

Figure 2 shows a comparison of the model predictions and observed data from the pilot study for post‐partum women representing the median lines and 95%PIs for dabigatran plasma concentration, aPTT and breast milk concentration, superimposed with the observed data from the pilot study. For plasma concentrations (Figure 2A) and aPTT (Figure 2B), these represent prospective predictions by the model, with the physiological and drug specific parameters implemented in the model as described above. Unfortunately, data from only one patient is available for aPTT. However, for breast milk concentration predictions and observed data (Figure 2C), this includes median line and 95%PI by the model and the observed data from the two patients included in the pilot study. This prediction cannot be considered as prospective since the parameter ${\mathrm{CL}}_{M}$ used was fitted to the observed data in the figure. Overall, with this limited data from the pilot study, the model captured the observed data adequately, which represents an initial and important (although limited) step towards validation of this model in this population.

**FIGURE 3:**
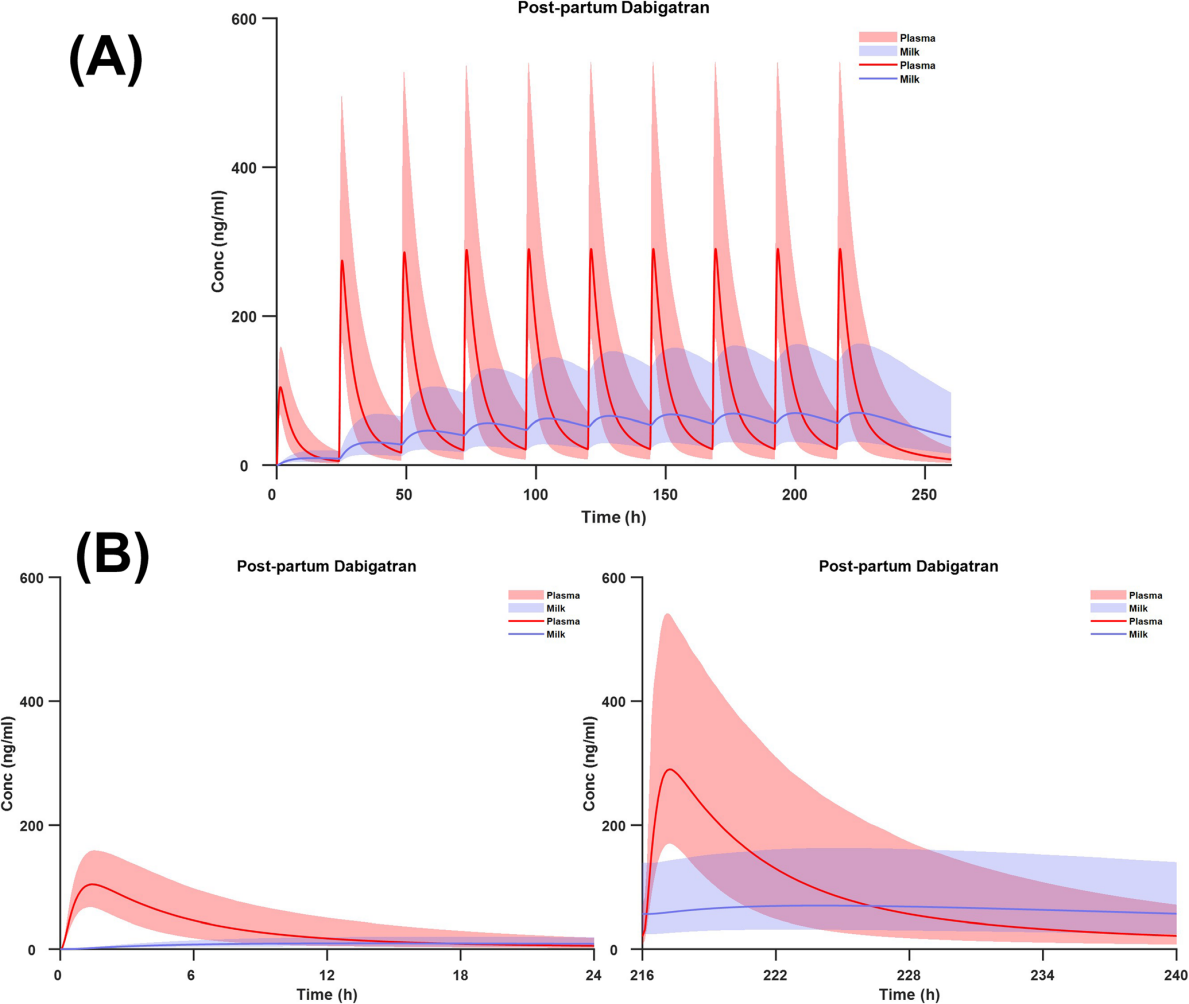
Simulated median lines and 95%PIs for plasma and breast milk concentrations in post‐partum women following multiple dosing of DABE.

Figure 3 shows the simulated median lines and 95%PI intervals for plasma and breast milk concentrations following 110 mg and 220 mg every 24 h for 10 days (Figure 3A). Figure 3B represents the profiles after the first dose and Figure 3C profiles after the last dose. These figures show that while plasma concentration reached steady‐state by the 5th dose, breast milk concentration appeared to reach steady‐state after this time. For plasma drug concentration this is consistent with the terminal half‐life of 12 – 17 h reported for dabigatran in the literature.^8^

Table 3 shows the summary of IDD and %RIDD simulated with two scenarios of single and multiple dosing assuming 150 or 200 mL/kg/day infant milk consumption. Consistent with the data from pilot study, a very low drug concentration and therefore very low IDD and %RIDD were obtained.

**TABLE 3:** A summary of infant daily dose (IDD ‐ μg), percentage relative infant daily dose (RIDD%) assuming 150 mL/kg/day and 200 ml/kg/day infant breast milk consumption.

Study	Dose	150 ml/kg/day		200 ml/kg/day	
		IDD (μg) – mean (SD)	RIDD (%) – mean (SD)	IDD (μg) – mean (SD)	RIDD (%) – mean (SD)
Single dose	220 mg	11.44 (4.78)	0.0039 (0.0016)	14.56 (6.09)	0.005 (0.0021)
Multiple dose	1st Dose (100 mg)	4.46 (1.71)	0.0015 (0.00058)	5.65 (2.14)	0.0019 (0.00073)
	2nd Dose (220 mg)	15.65 (6.57)	0.0053 (0.0022)	19.82 (8.24)	0.0068 (0.0028)
	Steady State (220 mg)	38.42 (17.35)	0.013 (0.0059)	48.72 (21.94)	0.017 (0.0075)

### 3.2 Proposed clinical study design and sample size calculations

For the safety arm, using a single dose of 220 mg (recommended adult dose) and assuming 200 mL/kg/day infant milk consumption, based on %RIDD ( mean=0.005%andsd=0.0021), the sample size calculation restricting the upper 95%CI to be below a safe threshold, ranging from as high as 0.02% was investigated; this value was considered highly conservative and close to the mean. A range of thresholds were explored because a safe threshold is not currently established for dabigatran. Also, it is important that resulting samples should contain an adequate number of subjects to obtain practical data of real relevance and credibility. As such, a sample size of n=20 for the safety arm of the study using a significance level of α=0.05 and power of 90% for a one‐sided test will ensure that the upper 95%CI should not cross a threshold of t=0.00569. The probability of achieving the proposed 95%CI width is set high at 0.9999.

For the efficacy arm, the power at 95%CI of $C_{\min}$ in post‐partum women greater than a threshold based on $C_{\min}$ in healthy non‐pregnant women was used to derive sample size. For post‐partum women the log transformed $C_{\min}$ ( mean−2.83ng/ml, sd−0.60) was compared to the $C_{\min}$ threshold of 9.87ng/ml ( 10th percentile and 2.29ng/ml in the log transformed domain). A sample size of between n=50 and 60 for this arm of the study using a significance level of α=0.05 and a power of 90% in a one‐sided test will ensure that the lower 95%CI should not cross a threshold of t=2.73−2.7415. The probability of achieving the proposed 95%CI width has been set to 0.9999. It is believed that this sample size will confidently provide clinically and practically relevant data.

## 4 DISCUSSION

In this study a PBPK model developed for DABE‐dabigatran and validated in healthy male volunteers^13^ was translated to healthy non‐pregnant, pregnant and post‐partum women, by accounting for key physiological parameter value changes that occur in these populations. The model was also used to explore the link between exposure and three coagulation indices of aPTT, DTT and ECT, based on established relationships that have been used for dose optimisation and licensing in other populations.^7^ In post‐partum women, the model was also empirically linked to breast milk concentration to obtain an integrated model. These models in different populations were finally used to explore PK and PD changes with the ultimate aim of designing future prospective clinical studies to assess safety and efficacy of DABE in post‐partum women.

The results showed that although there are significant changes in physiological parameters in the different populations examined, which were accounted for in the model, these changes do not result in significant differences in PK and PD between these populations. Also, a sensitivity analysis of the model in healthy male volunteers indicated sensitivity to key parameters to plasma concentration (Supplementary Material, Figure S4). The simulated median and PI were comparable, suggesting that the DABE dose requirement in post‐partum women is expected to be similar to healthy adult men and non‐pregnant women. Data from two patients from an earlier pilot study was used to verify model predictions in post‐partum women for dabigatran plasma and breast milk concentrations, and aPTT. Ideally, a natural progression for this work would be to further translate the model to neonates, this would allow an evaluation of the PK and PD of DABE‐dabigatran once consumed by infants during breastfeeding.^18^, ^20^ However, it has been shown that DABE concentration in plasma is very low; it is at least 50 times lower compared to dabigatran and it is only detected for a short period of time (below the detection limit mostly).^13^ Furthermore, the active drug dabigatran cannot be absorbed from the gastro‐intestinal tract or permeate mamillary epithelium due to its polar nature. Therefore, it is expected that neither DABE, nor dabigatran will be detectable in the plasma of infant following breastfeeding by mother on DABE. In support of such a notion, a study with tenofovir disoproxil fumarate, a prodrug with similar physicochemical properties to DABE, showed that very low concentrations of the active drug appear in breast milk and it is undetectable in the plasma of breastfed infants.^21^

The focus of the present study is on post‐partum women at risk of thromboembolism as dabigatran use in this population represents an unmet need. We propose a two‐arm clinical study, supported by the results of the modelling presented herein. The trial will be conducted in post‐partum women who have chosen not to breastfeed. In general, studies in post‐partum women are challenging and this explains why since the introduction of DOACs a quarter of a century ago, post‐partum women still do not have access to these drugs. Therefore, conducting trials evaluating the safety and efficacy of DABE in this population is important, otherwise women will continue to choose between breastfeeding and available treatment, which may not be the most effective, in terms of patient experience and satisfaction with treatment and also clinically or financially. The approach used in this study, has leveraged on an available model and data in the literature to design a future study in this special population. The safety arm will focus on the extent of dabigatran secretion in breast milk and as shown by the results presented herewith, this is expected to be very low. Also, as shown by the results of the simulations and the pilot study, the collection period for breast milk will be further extended beyond plasma collection time‐points to be able to characterise the kinetics fully. The safety arm of the clinical study will focus on PK and PD endpoints; plasma concentration and coagulation indices – aPTT, dTT and ECT data will be collected following dosing, and used for model verification and further optimisation of the model if necessary. The final model will therefore be used for dose optimisation in this population.

In conclusion, an integrated PBPK/PD model for DABE‐dabigatran in post‐partum women has been developed, which has been translated from published PBPK model for healthy men, exposure‐response models for coagulation indices in children and adults and available breast milk concentration data from a pilot study. This model was validated and subsequently used to design a future clinical study to evaluate safety and efficacy of DABE in postpartum women, which will provide supporting evidence for the use of this drug in this special population.

## AUTHOR CONTRIBUTIONS

Manuscript Writing: **Kayode Ogungbenro**, **Lorna Aucott**, **Farhad Kamali**, **Paul Ayuk**. Modelling: **Kayode Ogungbenro** Statistical Analysis: **Lorna Aucott** Conceptualisation: FK, PA.

## CONFLICT OF INTEREST STATEMENT

The authors declare no conflict of interest.

## Supporting information


**Table S1:** Percentage (%) of organ weights relative to total body weight for different populations (obtained from Valentin, 2002 [1]).
**Table S2:** Percentage of organ/tissue blood flows relative to total cardiac output for different populations (obtained from Valentin, 2002 [1]).
**Table S3:** A summary of drug specific parameters used for the DABE‐Dabigatran PBPK model in healthy men, healthy, pregnant and post‐partum women (parameters have been obtained with permission from Lang *et al.*, 2021 [2]).
**Figure S1:** Schematic representation of the compartmental model for empirical fitting of plasma and breast milk data from two individuals in pilot study [9].
**Table S4:** Parameter estimates obtained from fitting compartmental model to the plasma and breast milk data from two individuals in pilot study [9].
**Figure S2:** Simulated median plasma concentration, aPTT, DTT and ECT profiles in healthy men, healthy women, pregnant women, and post‐partum women.
**Figure S3:** Simulated plasma concentration, aPTT, DTT and ECT profiles (median and 95% prediction interval) in healthy and post‐partum women.
**Table S5:** Summary of the parameter estimates for PK/PD model for dabigatran plasma concentration and aPTT, a coagulation index surrogate (obtained from FDA, OCP Review, 2020 [4]).
**Table S6:** Summary of the parameter estimates for PK/PD model for dabigatran plasma concentration and DTT, a coagulation index surrogate (obtained from FDA, OCP Review, 2020 [4]).
**Table S7:** Summary of the parameter estimates for PK/PD model for dabigatran plasma concentration and ECT, a coagulation index surrogate (obtained from FDA, OCP Review, 2020 [4]).
**Figure S4:** Plots of absolute relative sensitivities (|RSven|) of the plasma concentration against time for some parameters of the PBPK model in healthy men.

## Data Availability

The data, model implementation and output generated in this study can be requested from the corresponding author, subject to reasonable access agreement.
