# Supplementary material for: Translation of a physiologically‐based pharmacokinetic model for dabigatran etexilate to the design of a safety and efficacy study in post‐partum women
Source: Br J Clin Pharmacol. 2025 Nov 21;92(4):1109–16. doi: 10.1002/bcp.70344 (PMC13021304; doi:10.1002/bcp.70344)
Supplement: Supplementary file 1 — Table S1: Percentage (%) of organ weights relative to total body weight for different populations (obtained from Valentin, 2002 [1]). Table S2: Percentage of organ/tissue blood flows relative to total cardiac output for different populations (obtained from Valentin, 2002 [1]). Table S3: A summary of drug specific parameters used for the DABE‐Dabigatran PBPK model in healthy men, healthy, pregnant and post‐partum women (parameters have been obtained with permission from Lang et al., 2021 [2]). Figure S1: Schematic representation of the compartmental model for empirical fitting of plasma and breast milk data from two individuals in pilot study [9]. Table S4: Parameter estimates obtained from fitting compartmental model to the plasma and breast milk data from two individuals in pilot study [9]. Figure S2: Simulated median plasma concentration, aPTT, DTT and ECT profiles in healthy men, healthy women, pregnant women, and post‐partum women. Figure S3: Simulated plasma concentration, aPTT, DTT and ECT profiles (median and 95% prediction interval) in healthy and post‐partum women. Table S5: Summary of the parameter estimates for PK/PD model for dabigatran plasma concentration and aPTT, a coagulation index surrogate (obtained from FDA, OCP Review, 2020 [4]). Table S6: Summary of the parameter estimates for PK/PD model for dabigatran plasma concentration and DTT, a coagulation index surrogate (obtained from FDA, OCP Review, 2020 [4]). Table S7: Summary of the parameter estimates for PK/PD model for dabigatran plasma concentration and ECT, a coagulation index surrogate (obtained from FDA, OCP Review, 2020 [4]). Figure S4: Plots of absolute relative sensitivities (|RSven|) of the plasma concentration against time for some parameters of the PBPK model in healthy men. [file BCP-92-1109-s001.docx]

**Supplementary Materials**

**Title: Translation of a physiologically-based pharmacokinetic model for dabigatran etexilate to the design of a safety and efficacy study in post-partum women**

**Running title: DABE PBPK model in post-partum women**

Kayode Ogungbenro, PhD^1^, Lorna Aucott, PhD^2^, Farhad Kamali, PhD^3,4^, and Paul Ayuk, PhD^3,4^

^1^Centre for Applied Pharmacokinetic Research

Division of Pharmacy and Optometry, School of Health Sciences

Faculty of Biology, Medicine and Health

University of Manchester, Manchester, M13 9PT

United Kingdom

^2^Insititute of Medical Sciences, University of Aberdeen

^3^Translational and Clinical Research Institute, Newcastle University, Newcastle University, Newcastle upon Tyne, UK

^4^Newcastle upon Tyne Hospitals NHS Foundation Trust, UK

Correspondence to

K Ogungbenro, Tel: +44 161 275 2399, Email: [kayode.ogungbenro@manchester.ac.uk](mailto:kayode.ogungbenro@manchester.ac.uk)

Keywords: dabigatran, post-partum women, pharmacokinetics, modelling

Table S1: Percentage (%) of organ weights relative to total body weight for different populations (obtained from Valentin, 2002 [1]).

| Organ/Tissue | Healthy men | Healthy women | Pregnant women | Post-partum women |
| --- | --- | --- | --- | --- |
| Lungs | 1.46 | 1.4 | 1.16 | 1.27 |
| Muscle | 40.5 | 29.17 | 24.14 | 26.48 |
| Adipose | 22.8 | 30 | 24.83 | 27.23 |
| Skin | 4.36 | 3.83 | 3.17 | 3.48 |
| Heart | 0.47 | 0.42 | 0.34 | 0.38 |
| Brain | 2.03 | 2.17 | 1.79 | 1.97 |
| Kidney | 0.43 | 0.46 | 0.38 | 0.42 |
| Bones | 11.8 | 10.4 | 8.61 | 9.44 |
| Spleen | 0.21 | 0.22 | 0.18 | 0.20 |
| Pancreas | 0.20 | 0.20 | 0.17 | 0.18 |
| Intestine | 1.42 | 1.67 | 1.37 | 1.50 |
| Stomach | 0.21 | 0.23 | 0.19 | 0.21 |
| Liver | 2.42 | 2.33 | 1.93 | 2.12 |
| Portal vein | 0.014 | 0.014 | 0.012 | 0.013 |
| Hepatic artery | 0.007 | 0.007 | 0.0058 | 0.0064 |
| Uterus/Foetus | - | 0.13 | 8.14^*^ | 1.45 |

^*^Includes foetus, placenta and amniotic fluid

Table S2: Percentage of organ/tissue blood flows relative to total cardiac output for different populations (obtained from Valentin, 2002 [1]).

| Organ/Tissue | Healthy men | Healthy women | Pregnant women | Post-partum women |
| --- | --- | --- | --- | --- |
| Muscle | 17 | 12 | 8.8 | 10 |
| Adipose | 5 | 8.50 | 7.8 | 8.9 |
| Skin | 5 | 5 | 8.7 | 9.9 |
| Heart | 4 | 5 | 3.7 | 4.2 |
| Brain | 12 | 12 | 8.8 | 10.0 |
| Kidney | 19 | 17 | 16.6 | 19 |
| Bones | 5 | 5 | 3.7 | 4.2 |
| Spleen | 3 | 3 | 2.2 | 2.5 |
| Pancreas | 1 | 1 | 0.7 | 0.8 |
| Intestine | 10 | 11 | 11 | 12.6 |
| Stomach | 1 | 1 | 1 | 1.1 |
| Hepatic vein | 25 | 27 | 20 | 22.8 |
| Enterocyte | 5 | 5 | 5 | 5.7 |
| Uterus/Foetus | - | 0.4 | 12 | 0.4 |

^*^Includes foetus and placenta

Table S3: A summary of drug specific parameters used for the DABE-Dabigatran PBPK model in healthy men, healthy, pregnant and post-partum women (parameters have been obtained with permission from Lang *et. al.*, 2021 [2])

| **Category** | **Parameter** | **Definition** | **Unit** | **Initial value** | **Reference/Source** |
| --- | --- | --- | --- | --- | --- |
| **Physiological** | Vsys | Systemic blood volume | L | 2.37 | Valentin, 2002 [1] |
|  | Qliv | Liver blood flow | L/h | 97.5 | Valentin, 2002 [1] |
|  | Vliv | Liver tissue volume | L | 1.69 | Valentin, 2002 [1] |
|  | TMEPPI | Population total membrane protein | mg/g small intestine | 2737 | Simcyp [3] |
|  | AP-gp | P-gp abundance (jejunum) | pmol/mg membrane protein | 0.4 | Simcyp [3] |
|  | RelP-gp | Relative P-gp abundance in duodenum and  ileum relative to jejunum | | 0.51/1/1.51 | Simcyp [3] |
| **Drug-specific** | MW | Molecular weight | g/mol | 471.51 | FDA, 2020 [4] |
| **Dabigatran** | pKa | Acid dissociation constant |  | basic – 12.4, acidic – 4.4/4.1 | FDA, 2020 [4] |
|  | logP | Logarithm of partition coefficient |  | -2.2 | FDA, 2020 [4] |
|  | fu,p | Fraction unbound in plasma |  | 0.65 | Moj et al., 2019 [5] |
|  | BP | Blood-to-plasma ratio |  | 0.67 | Moj et al., 2019 [6] |
|  | Papp | Apparent permeability (Caco-2) | 10^-6^ cm/s | 0.35 | FDA, 2020 [4] |
|  | Qgut | Gut blood flow | L/h | 2.23 | Lang et al., 2021 [2] |
|  | KbLi | Blood-to-liver partition coefficient |  | 1.49 | Lang et al., 2021 [2] |
|  | KbKN | Blood-to-kidney partition coefficient |  | 1.39 | Lang et al., 2021 [2] |
|  | Kper | Peripheral rate | 1/h | 36.6 | Lang et al., 2021 [2] |
|  | Vper,EC | Extracellular volume of peripheral compartment |  | 7.85 | Lang et al., 2021 [2] |
|  | Vper,IC | Intracellular volume of peripheral compartment |  | 52.6 | Lang et al., 2021 [2] |
|  | KbperEC | Blood-to-peripheral (extracellular) partition coefficient |  | 0.813 | Lang et al., [2] |
|  | KbperIC | Blood-to-peripheral (intracellular) partition coefficient |  | 0.76 | PK-Sim [6] |
|  | Pdiff | Passive diffusion between extracellular and intracellular matrices | 10^-6^ cm/s | 0.56 | Lang et al., 2021 [2] |
|  | Saper | Surface area of the peripheral compartment | dm^2^ | 29866 | PK-Sim [6] |
|  | CLr | Renal blood clearance | L/h | 10.8 | FDA, 2020 [4] |
|  | CLh | Hepatic blood clearance | L/h | 2.7 | FDA, 2020 [4] |

Table S3 cont’d

| **Category** | **Parameter** | **Definition** | **Unit** | **Initial value** | **Reference/Source** |
| --- | --- | --- | --- | --- | --- |
| **Dabigatran** | MW | Molecular weight | g/mol | 627.75 | FDA, 2020 [4] |
| **etexilate** | pKa | Acid dissociation constant |  | basic – 4/6.7 | FDA, 2020 [4] |
|  | logP | Logarithm of partition coefficient |  | 3.8 | FDA, 2020 [4] |
|  | fu,p | Fraction unbound in plasma |  | 0.07 | Moj et al., 2019 [5] |
|  | BP | Blood-to-plasma ratio |  | 3 | Moj et al., 2019 [5] |
|  | fu,gut | fraction unbound in the gut |  | 1 | Lang et al., 2021 [2] |
|  | Papp | Apparent permeability (Caco-2) | 10^-6^ cm/s | 14.4 | FDA, 2020 [4] |
|  | ka | Absorption rate | 1/h | 9.1-14.4 | Lang et al., 2021 [2] |
|  | Qgut | Gut blood flow | L/h | 16.5 | Lang et al., 2021 [2] |
|  | KbLi | Blood-to-liver partition coefficient |  | 0.085 | Lang et al., 2021 [2] |
|  | Qper | Peripheral blood flow | L/h | 97.5 | Valentin, 2002 [1] |
|  | Vper | Peripheral volume | L | 60.7 | Valentin, 2002 [1] |
|  | Kbper | Blood-to-peripheral partition coefficient |  | 13.4 | Lang et al., 2021 [2] |
|  | Vmax,CES1 | CES1 maximum velocity | pmol/min/mg protein | 6860 | Lang et al., 2021 [2] |
|  | Km,CES1 | CES1 Michaelis-Menten constant | µM | 33.5 | Laizure et al., 2013 [7] |
|  | Vmax,CES2 | CES2 maximum velocity | pmol/min/mg protein | 30.8 | Laizure et al., 2013 [7] |
|  | Km,CES2 | CES2 Michaelis-Menten constant | µM | 5.5 | Laizure et al., 2013 [7] |
|  | Vmax,P-gp | P-gp maximum velocity | pmol/min/pmol P-gp | 44600 | Lang et al., 2021 [2] |
|  | Km,P-gp | P-gp Michaelis-Menten constant | µM | 2.6 | Yamazaki et al., 2019 [8] |

# PBPK/Breast Milk model

Figure S1: Schematic representation of the compartmental model for empirical fitting of plasma and breast milk data from two individuals in pilot study [9].

Table S4: Parameter estimates obtained from fitting compartmental model to the plasma and breast milk data from two individuals in pilot study [9].

| **Parameter** | **Description** | **Unit** | **Estimate** | **%RSE** |
| --- | --- | --- | --- | --- |
| ka | Absorption rate constant | /h | 0.564 | 59 |
| CL | Clearance | L/h | 77.4 | 14 |
| V | Volume of distribution | L | 525 | 35 |
| CL_M_ | Clearance secretion/reuptake between breast milk and blood | L/h | 0.0126 | 34 |
| V_M_ | Milk volume | L | 0.5 FIXED | - |
| Plasma (prop) | Residual variability | % | 34 | 43 |
| Plasma (add) | Residual variability | ng/ml | 0.01 FIXED | - |
| Milk (prop) | Residual variability | % | 98 | 50 |
| Milk (add) | Residual variability | ng/ml | 0.01 FIXED | - |

Figure S2: Simulated median plasma concentration, aPTT, DTT and ECT profiles in healthy men, healthy women, pregnant women, and post-partum women.

Figure S3: Simulated plasma concentration, aPTT, DTT and ECT profiles (median and 95% prediction interval) in healthy and post-partum women.

# PBPK/PD model simulations

## Activated partial thromboplastin time (aPTT),

The PK/PD models for coagulation indices; activated partial thromboplastin time (aPTT), diluted thrombin time (dTT) and ecarin clotting time (ECT) were used to explore exposure-response changes in different population groups. These relationships were obtained from the FDA Office Clinical Pharmacology (FDA, OCP) review document [4]. The relationships have been established from different dose levels in adults and children.

For aPTT, the PK/PD model was described using a non-linear Emax function, which has an age effect on the baseline on baseline. The parameter estimates use for simulation are as follows in Table S5.

1. Table S5: Summary of the parameter estimates for PK/PD model for dabigatran plasma concentration and aPTT, a coagulation index surrogate (obtained from FDA, OCP Review, 2020 [4]).

| **Parameter** | **Unit** | **Typical individual Value** | **Between-subject variability (%)** |
| --- | --- | --- | --- |
| Baseline | sec | 36.1 | - |
| Baseline (<5.8 months) | sec | 44.8 |  |
| Age on baseline | months | 5.80 |  |
| EC50 | ng/ml | 3.68 | 28.5 |
| Emax | - | 2.02 | 74.9 |
| Corr Baseline-Emax | - | - | -0.722 |
| Residual variability (proportional) | - | - | 17.8 |

## Diluted thrombin time (dTT)

For dTT, the PK/PD relationship was described by a linear function, with no covariate effect on the parameters. The parameters of this model are therefore as follows in Table S6.

1. Table S6: Summary of the parameter estimates for PK/PD model for dabigatran plasma concentration and DTT, a coagulation index surrogate (obtained from FDA, OCP Review, 2020 [4]).

| Parameter | Unit | Typical Individual Value | Between subject variability (%) |
| --- | --- | --- | --- |
| Baseline | sec | 32.1 | 6.70 |
| Slope | /(ng/ml) | 0.00373 | 30.1 |
| Corr Baseline-Slope | - | - | -0.646 |
| Residual variability (proportional) | - | - | 10.7 |

## Ecarin clotting time (ECT)

1. For ECT, the PK/PD relationship was described by a linear function, with covariate effect of age on baseline and slope on the parameters. The parameters of this model are therefore as follows in Table S7.
2. Table S7: Summary of the parameter estimates for PK/PD model for dabigatran plasma concentration and ECT, a coagulation index surrogate (obtained from FDA, OCP Review, 2020 [4]).

| Parameter | Unit | Typical Individual Value | Between subject variability (%) |
| --- | --- | --- | --- |
| Baseline | sec | 36.4 | 19.9 |
| Baseline (<5.8 months) | sec | 39.9 |  |
| Age on Baseline | months | 5.80 |  |
| Slope | /(ng/ml) | 0.00732 | 45.0 |
| Age on slope | - | -0.0633 |  |
| Corr Baseline-Slope | - | - | -0.888 |
| Residual variability (proportional) | - | - | 11.4 |

## Sensitivity analysis of the PBPK in healthy male volunteers

A sensitivity analysis of dabigatran PBPK model in healthy male volunteers was conducted by calculating the sensitivity (Jacobian matrix) of the plasma concentration relative to parameters of the model. The parameters used for this analysis included, cardiac output, total body weight, renal clearance, hepatic clearance, fraction unbound in plasma and blood-to-plasma ratio. Wendling *et al*., [10] described in details the method used for this analysis. The results in Figure S4 showed plots of relative sensitivity coefficient of venous plasma concentration (|RS_ven_|) for the parameters against time. Based on the widely used value of 0.1, all the parameters showed significant influence on the venous plasma concentration over the range of simulated time.

Figure S4: Plots of absolute relative sensitivities (|RSven|) of the plasma concentration against time for some parameters of the PBPK model in healthy men.


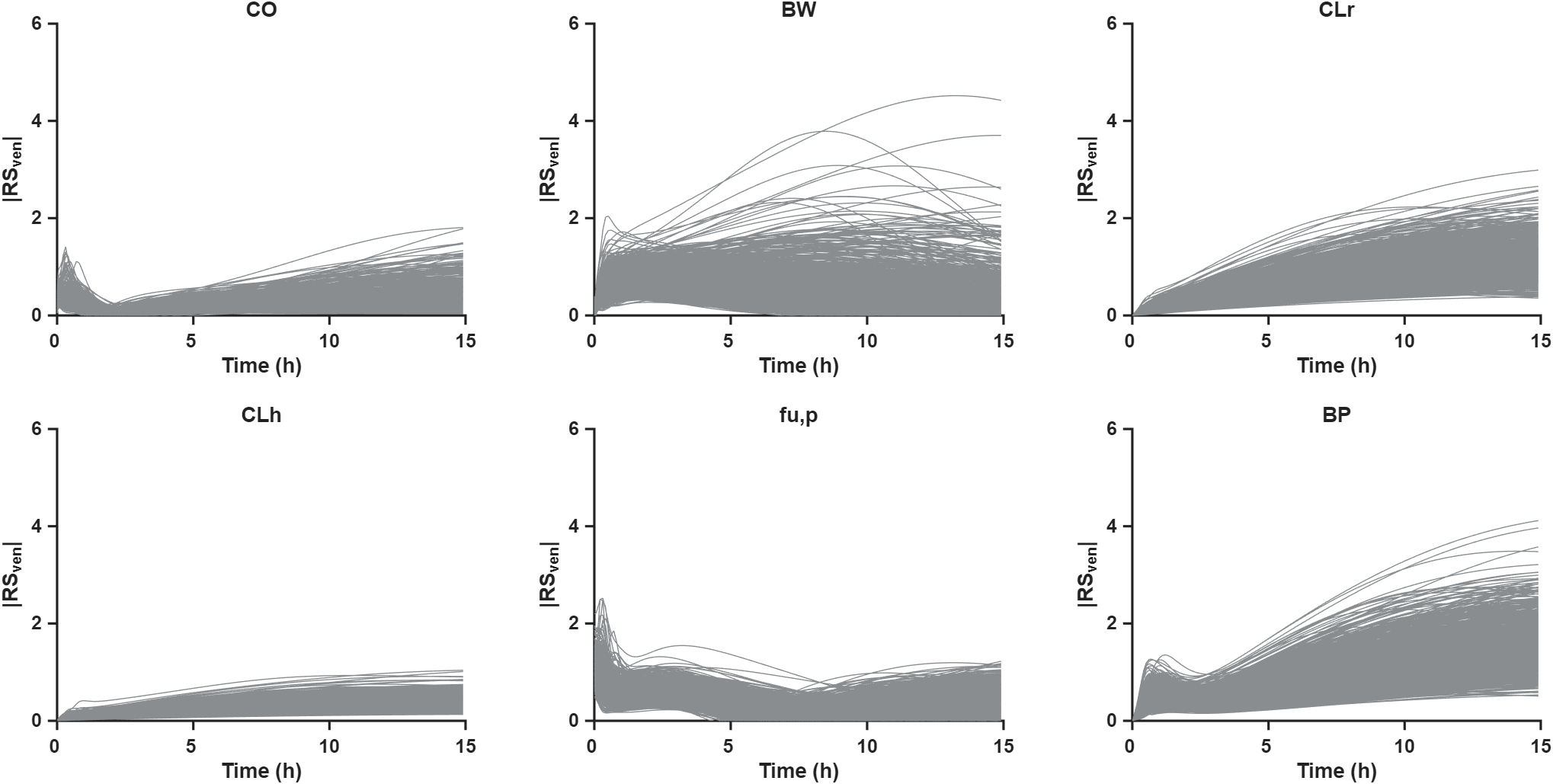


CO=cardiac output, BW=total body weight, CLr=renal clearance, CLh=hepatic clearance, fu,p= fraction unbound in plasma, BP=blood-to-plasma ratio

References

1. Valentin J. Basic anatomical and physiological data for use in radiological protection: reference values: ICRP Publication 89. Ann ICRP 2002; 32: 1-277.

2. Lang J, Vincent L, Chenel M, Ogungbenro K, Galetin A. Reduced physiologically-based pharmacokinetic model of dabigatran etexilate-dabigatran and its application for prediction of intestinal P-gp-mediated drug-drug interactions. Eur J Pharm Sci 2021; 165: 105932.

3. Simcyp™ PBPK Simulator (version 21; Certara, Sheffield, UK). In.

4. FDA(OCP). Pradaxa (Dabigatran etexilate) - Clinical pharmacology/toxicology NDA review and evaluation. In, 2020.

5. Moj D, Maas H, Schaeftlein A, Hanke N, Gómez-Mantilla JD, Lehr T. A Comprehensive Whole-Body Physiologically Based Pharmacokinetic Model of Dabigatran Etexilate, Dabigatran and Dabigatran Glucuronide in Healthy Adults and Renally Impaired Patients. Clin Pharmacokinet 2019; 58: 1577-93.

6. PK-Sim, 2018. PK-Sim open systems pharmacology suite documentation.

7. Laizure SC, Parker RB, Herring VL, Hu Z-Y. Identification of Carboxylesterase-Dependent Dabigatran Etexilate Hydrolysis. Drug Metab Dispos 2014; 42: 201-06.

8. Yamazaki S, Costales C, Lazzaro S, Eatemadpour S, Kimoto E, Varma MV. Physiologically-Based Pharmacokinetic Modeling Approach to Predict Rifampin-Mediated Intestinal P-Glycoprotein Induction. CPT Pharmacometrics Syst Pharmacol 2019; 8: 634-42.

9. Ayuk P, Kampouraki E, Truemann A, Sidgwick F, McDonald L, Bingham J, Murphy P, Kamali F. Investigation of dabigatran secretion into breast milk: Implications for oral thromboprophylaxis in post-partum women. Am J Hematol 2020; 95: E10-E13.

10. Wendling T, Dumitras S, Ogungbenro K, Aarons L. Application of a Bayesian approach to physiological modelling of mavoglurant population pharmacokinetics. J Pharmacokinet Pharmacodyn 2015; 42: 639-57.
